# Translation and validation of the Brazilian Portuguese version of the quality of life vascular access device questionnaire for chemotherapy patients

**DOI:** 10.31744/einstein_journal/2025AO1665

**Published:** 2025-07-23

**Authors:** Bruno Jeronimo Ponte, Carolina Carvalho Jansen Sorbello, Ricardo Ferreira Mendes de Oliveira, Maria Fernanda Cassino Portugal, Andressa Cristina Sposato Louzada, Marcelo Fiorelli Alexandrino da Silva, Lucas Lembrança Pinheiro, Cynthia de Almeida Mendes, Nelson Wolosker

**Affiliations:** 1 Hospital Municipal da Vila Santa Catarina Dr. Gilson de Cássia Marques de Carvalho Hospital Israelita Albert Einstein São Paulo SP Brazil Hospital Municipal da Vila Santa Catarina Dr. Gilson de Cássia Marques de Carvalho; Hospital Israelita Albert Einstein, São Paulo, SP, Brazil.; 2 Hospital Israelita Albert Einstein Faculdade Israelita de Ciências da Saúde Albert Einstein São Paulo SP Brazil Faculdade Israelita de Ciências da Saúde Albert Einstein, Hospital Israelita Albert Einstein, São Paulo, SP, Brazil.; 3 Universidade de São Paulo Faculdade de Medicina São Paulo SP Brazil Faculdade de Medicina, Universidade de São Paulo, São Paulo, SP, Brazil.

**Keywords:** Translations, Vascular access devices, Quality of life, Validation studies as topic, Surveys and questionnaires

## Abstract

**Background:**

The use of long-term devices for chemotherapy in neoplastic diseases is very common. The CAVA trial was an extensive study that prospectively evaluated more than 1000 patients undergoing chemotherapy and randomly allocated them to three groups based on the type of catheters used: peripherally inserted central catheters, Port, or Hickmann. For this study, the Quality of Life Vascular Access Device (QoLVAD) questionnaire was administered to assess the impact of these devices (abovementioned catheters) on the patients’ daily lives. routine However, no Brazilian Portuguese version of the questionnaire exists, hindering its use among Brazilian patients.

**Objective:**

To translate the CAVA trial QoLVAD to Brazilian Portuguese and validate it so that it can be used in the Brazilian population.

**Methods:**

A total of 167 patients with long term vascular access devices for chemotherapy participated in the study. After translation and retranslation, construct validity was analyzed by identifying the correlation between QoLVAD and the European Quality of Life Questionnaire (EuroQol). To determine the reliability, internal consistency and test-retest analysis with at least a 7-day interval between two administrations of the questionnaire were calculated.

**Results:**

The results revealed good internal and external consistency of the QoLVAD. Significant correlations were found between the QoLVAD and EuroQol (r −0,658 and p<0.001).

**Conclusion:**

The Brazilian Portuguese version of the QoLVAD exhibited good clinimetric properties and proved to be applicable to the Brazilian population.

## In Brief

Long-term vascular access devices for chemotherapy are essential. This study aimed to translate and validate the Quality of Life Vascular Access Device (QoLVAD) questionnaire from English to Brazilian Portuguese. Quality of Life Vascular Access Device was translated to Brazilian Portuguese by the authors. The questionnaire was then administered to patients at two different times to evaluate its internal and external consistency. Its validity and construct analysis were established through comparison with EuroQol (5D-5L). Internal and external consistency were also confirmed. Its significant correlations with EuroQol demonstrated that QoLVAD can be applied to the Brazilian population.

## Highlights

■ Assessing how vascular access devices affect quality of life is essential to effectively manage chemotherapy patients.
■ The translation and validation of the QoLVAD questionnaire demonstrated that the Brazilian Portuguese version can be applied to the Brazilian population.

## Graphical abstract

**Figure:**
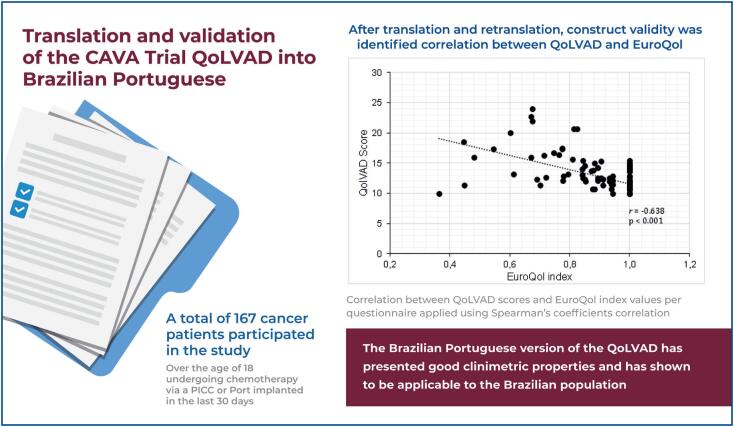

## INTRODUCTION

The use of long-term vascular devices for chemotherapy in cancer treatments is widespread.^(1)^ The National Cancer Institute estimated that there would be approximately 704,000 new cancer cases in Brazil by 2023.^(2)^

Intravenous chemotherapy can be administered through either peripheral or dedicated vascular access.^(3)^ The latter includes peripherally inserted central catheters (PICCs), partially implantable tunneled long-term central catheters (Hickmann), and fully implantable tunneled central catheters (Port).^(4)^ These devices are particularly beneficial for long-term intravenous therapy because they deliver medication directly into central veins, thereby reducing the risk of complications, such as phlebitis and extravasation, that can occur in smaller veins when using vesicant chemotherapy drugs.^(5)^

While numerous studies have examined adverse outcomes of catheter types, literature regarding the quality of life related to each catheter modality is lacking.^(6-9)^

The CAVA trial was a comprehensive study that prospectively evaluated over 1,000 patients undergoing chemotherapy.^(10)^ These patients were randomly assigned to three groups based on the type of venous access device they used: PICC, Port, or Hickmann catheters.^(5,10)^ The authors found that patients using Port devices experienced fewer catheter-related complications.^(5)^ To assess the impact of these devices on patients’ daily lives, the study utilized a specific questionnaire called the Quality of Life Questionnaire Vascular Access Device (QoLVAD) questionnaire. An evaluation of the psychosocial aspects related to the catheters used by CAVA trial patients revealed a preference for Port catheters. However, this preference was not universal among all participants.^(11)^

Another study focusing exclusively on patients using PICC devices revealed that most patients had positive experiences, as the devices did not affect their quality of life.^(12)^ Additionally, a second study focusing specifically on patients with PICCs and categorizing them based on the type of primary tumor concluded that the device had minimal effects on patients’ quality of life. Most issues reported were related to the underlying disease rather than the catheter itself.^(13)^

In a prospective cohort of 35 patients receiving brachial insertion for PICC catheters, 94.3% of respondents noted that they would recommend the device to others. Most patients reported no harm to their daily routines or feelings of anxiety about the device.^(14)^

High-quality data from randomized studies with high statistical power are essential for guiding clinical practices and decisions regarding vascular access in long-term intravenous therapies. However, a translated and validated tool in Brazilian Portuguese to assess the quality of life for patients with long-term catheters is currently lacking.

## OBJECTIVE

The goal of this study was to translate and validate the Quality of Life Vascular Access Device used by the CAVA trial research group into Brazilian Portuguese.

## METHODS

The research was conducted among patients undergoing chemotherapy through long-term venous access during their cancer follow-up at a tertiary oncological center.

This study complied with the principles outlined in the Helsinki Declaration and received approval from the Ethics Committee *of Hospital Israelita Albert Einstein* (CAAE: 59982622.3.0000.0071; #5.511.376). Before participating, all patients received comprehensive information about the study's objectives and procedures and provided their informed consent.

All the collected data were handled confidentially and stripped of any identifying information.

### Translation

The standard "forward-backward" method was used to translate the questionnaire developed by the authors of the CAVA trial from English to Brazilian Portuguese. Two health professionals who were fluent in English but were native Brazilian Portuguese speakers were selected for the task. They translated the questionnaire items and answers to create a provisional version. This initial version was then tested on two voluntary patients and adjustments were made before the re-translation process.

Next, two independent professionals with the same qualifications re-translated the provisional version back into English in order to assess its compatibility with the original English version. The re-translated questionnaire was then culturally adapted and renamed the Revised Provisional Version.

Then, two members of the research group, both vascular surgeons, checked the Revised Provisional Version to ensure it effectively assessed the necessary elements for analyzing patients’ quality of life when using catheters for chemotherapy. The researchers compared the various versions with the original text, identifying and correcting any discrepancies. A consensus version was then formulated, with careful attention paid to preserving semantic equivalence and ensuring that the vocabulary was simple and direct (Supplementary Material - *Versão em Português*).

### Sample

A convenience sample of 180 patients of both sexes were recruited for this study, which was conducted in an oncological outpatient setting. The inclusion criteria were patients over the age of 18 undergoing chemotherapy via a PICC or Port catheter implanted in the last 30 days and who agreed to participate by signing an informed consent form. Exclusions were made for patients under 18 years of age, those who had been using venous devices for less than 30 days prior to the administration of the questionnaire, patients who died between questionnaire re-administrations, and those who underwent catheter changes within that time frame.

Of the 180 patients invited to participate in the study, 167 met the appropriate clinical criteria and accepted to be included in the study.

The included participants were asked about their quality of life related to the vascular devices they were currently using—PICC or Port. Data were collected using two assessment tools: the QoLVAD and the European Quality of Life Questionnaire (EuroQol), which comprises five domains with five response options indicating the levels of severity per question (EQ-5D-5L).

### Validation

To analyze the validity of the QoLVAD construct, we compared data obtained from its 16 questions with the EQ-5D-5L index value. Data from the QoLVAD questionnaire were collected through interviews conducted by a single examiner. To determine the final QoLVAD score, the average of the responses was calculated and multiplied by 10, resulting in scores ranging from 10 to 40, with higher scores indicating patients’ poorer quality of life.

The EuroQol has been translated into Brazilian Portuguese.^(15)^ Each country has a predefined value set used to calculate its final index score—based on how its population values different health states—making it possible to highlight the differences between countries. Since a value set for the EQ-5D-5L is not yet available in Brazil, we calculated the index score using the value from the United States, as recommended by the EuroQol organization.^(16)^ In contrast to QoLVAD, the EuroQol index score ranges from a maximum of 1, indicating the best quality of life, to lower numbers, representing poor quality of life.

The patients responded to all 16 questions of the QoLVAD questionnaire. Their answers ranged from 1 (does not affect quality of life) to 4 (affects it a lot). A non-apply category (0) was included, as certain questions did not pertain to all patients (*e.g*., regarding difficulties driving a car, which is applicable only if the patient drives).

### Internal consistency

To analyze the reliability of the QoLVAD, a sub-sample of 46 patients was interviewed on two different occasions, with a minimum interval of 7 days and a maximum interval of 30 days. At both times, the interviews were conducted by the same evaluator.

Inter-observer analysis was assessed by interviewing 31 patients, with the QoLVAD being applied twice—at different times and by two different observers—within a maximum interval of 7 days.

### Statistical analysis

The demographic analysis was conducted by calculating the means and medians of the assessed parameters: age, sex, and body mass index. The association between QoLVAD and EQ-5D-5L was analyzed using Spearman's rank correlation coefficient. A coefficient greater than 0.5 was considered sufficient to validate the questionnaire, with a statistical significance set at p<0.05.

The qualitative variants from the questionnaire were compared using Kendall's Tau correlation coefficient for internal validation and inter-observer evaluation analysis. Coefficients below and above 0.5 were considered weak and moderate, respectively, with statistical significance also set at p<0.05.

## RESULTS

Among the 180 invited patients, 167 agreed to participate in the study. Table 1 presents the demographic characteristics of the interviewed patients. The distribution of men and women was approximately equal. Most patients were in their sixties and were not considered obese.

**Table 1:** Characteristics of the patients

Variables		Number/average	(%)
Sex			
	Male	88	52.70
	Female	79	47.30
Age (years)		56.94 ± 14.21 (19-86)	
Weight (kg)		65.95 ± 16.66 (35-145)	
Height (cm)		164.74 ± 9.34 (141-186)	
Body mass index (kg/m^2^)		24.26 ± 5.6 (13.49-45.25)	

**Figure 1:**
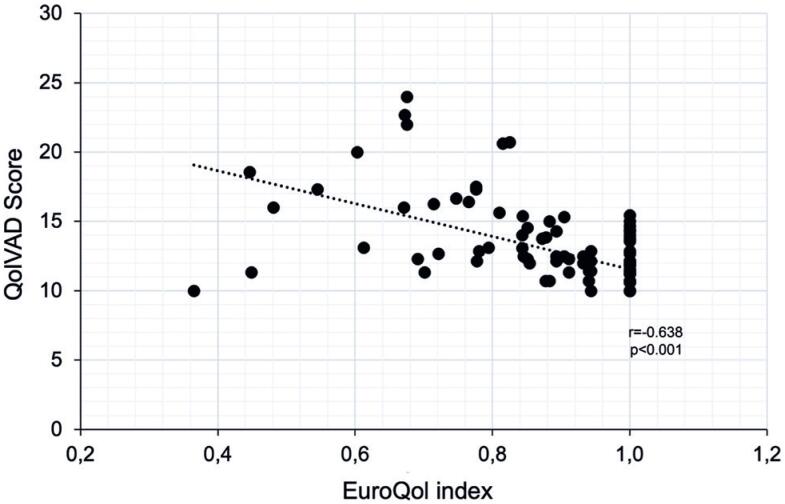
Scatter plot demonstrating the correlation between QoLVAD scores and EuroQol index values per questionnaire administered using Spearman's coefficients correlation

Figure 1 illustrates the Spearman correlation coefficients between the QoLVAD and EuroQol questionnaires. The negative coefficient can be attributed to the different parameters used by the two questionnaires. For example, higher scores indicate a poorer quality of life in QoLVAD but a better quality of life in EuroQol. The correlation was found to be negative and statistically significant with a coefficient of r=-0.658 (p<0.001).

Table 2 presents data on the reliability of the QoLVAD questionnaire. The results reflect patients responses on two separate occasions, which were analyzed using Kendall's Tau B correlation test. Questions 1, 2, 3, and 8 exhibited no variability in responses, resulting in an absolute agreement between the two moments the QoLVAD questionnaire was administered. Although a total of 46 patients completed the questionnaires, only 26 responded to question 9, which limited the significance of the analysis. All other questions demonstrated a moderate correlation and were statistically significant.

**Table 2:** Internal validation using Kendall's Tau rank correlation coefficient

QoLVAD question	Correlation	p value
1	1	*
2	1	*
3	1	*
4	0.609	<0.001
5	0.699	<0.001
6	0.989	<0.001
7	1	<0.001
8	1	*
9	0.348	0.082
10	0.564	<0.001
11	0.656	<0.001
12	1	<0.001
13	1	<0.001
14	0.5	<0.001
15	0.627	<0.001
16	0.693	<0.001

* Absolute agreement between the questionnaires administered by the same observer.

For the inter-observer analysis, 31 out of the 167 patients were interviewed by two observers at different times. Statistical analysis was conducted using Kendall's Tau correlation coefficient owing to the small sample size and minimal variability in the responses. The results are presented in table 3. All questions demonstrated positive correlations. Specifically, Questions 1, 2, 3, 5, 8, 9, 10, 11, and 13 yielded the same answers with both observers, resulting in absolute agreement. Although Question 16 was not statistically significant, it still exhibited a positive correlation.

**Table 3:** Inter-observer validation comparing questionnaires using Kendall's Tau rank correlation coefficient

QoLVAD question	Correlation	p value
1	1	*
2	1	*
3	1	*
4	0.462	0.016
5	1	*
6	0.822	<0.001
7	0.631	<0.001
8	1	*
9	1	*
10	1	*
11	1	*
12	1	<0.001
13	1	*
14	0.522	0.001
15	0.694	<0.001
16	0.334	0.062

* Absolute agreement between the questionnaires administered by the different observers.

## DISCUSSION

This study aimed to translate and validate the QoLVAD questionnaire for use in a Brazilian sample. The objective was to assess the quality of life in patients using long-term catheters for chemotherapy.

The QoLVAD questionnaire was developed by the authors of the CAVA trial to specifically assess changes in the quality of life caused by PICC, Port, and Hickmann catheters in patients undergoing chemotherapy.^(5,10)^

The original English version of the QoLVAD was translated to Brazilian Portuguese using the forward-backward methodology.^(17,18)^ Few changes were made to the questionnaire to ensure cultural appropriateness, as some items did not apply to the Brazilian context. When assessing patients’ work performance, individuals engaging in domestic activities we included, as many patients were not working due to their cancer diagnosis. Additionally, for the last QoLVAD question, the impact of PICC catheters on the frequency of hospital visits for changing PICC-related dressings was also considered.

Several widely recognized questionnaires assess general quality of life, with the most frequently used being the 36-item Short Form Survey and the EuroQol, both of which have already been translated into and validated in Portuguese.^(15)^ To conduct the analysis for external validation, the QoLVAD and EuroQol questionnaires were compared. The EuroQol was chosen because it has a well-documented Portuguese version and is concise and easy to understand.^(15)^

Analyzing the two questionnaires involved comparing the average value of the QoLVAD answers with the value of the EuroQol index. As Brazil does not currently have the set of values needed to calculate the EQ-5D-5L index, the set of values established by the United States was used, as recommended by EuroQol.^(16,19)^ A negative coefficient was found, corroborating the construct analysis between the two questionnaires.

To validate inter-observer reliability, patients were interviewed by two different observers who had previously been trained for the assessment process. The questionnaires were compared using Kendall's Tau-b, which demonstrated that the questions on the QoLVAD were positively consistent between the different observers. Question 16—which evaluates aspects of the quality of life of patients using catheters—was the only one that was not statistically significant, with a poor but positive correlation. We believe that the different times of survey administration may have influenced the results on this subjective question.

The internal validation analysis demonstrated a positive correlation between the responses the same observer at different times. All the questions demonstrated a positive correlation. Question 9 addresses how the use of vascular access affects patients jobs. As many of the institution's cancer patients remained absent from work because of their cancer treatment, several participants skipped this question. Moreover, some patients stopped working because of their treatment between this study's interviews, providing different answers when the questionnaire was administered at different times.

Some limitations were encountered in validating the QoLVAD. Many patients did not answer specific QoLVAD questions. In particular, questions about driving, using public transport, shopping, working, or engaging in physical activities were often left unanswered, as some patients did not participate in these activities. Consequently, these questions were categorized as "does not apply"—a designation not included in the original questionnaire, leading to reduced sample size for statistical comparison (Question 9 in internal validation). Additionally, some patients experienced changes in their functional status and activities of daily living because of cancer and/or its treatment, which further impacted the statistical analysis in terms of varying scores on the questionnaires at different times (Question 16 on inter-observer reliability).

Furthermore, external validation was statistically weakened by comparing an established score to the QoLVAD questionnaire, which was not described by the authors of the CAVA trial.

These results reveal that the translation and validation process of the QoLVAD questionnaire into Brazilian Portuguese was satisfactory. It was found to have a statistically significant correlation with the EuroQol (construct validation) and good reproducibility indices. Hence, it can be concluded that the Brazilian Portuguese version of the QoLVAD can be used in clinical practice to assess the impact of different vascular devices for chemotherapy on patients’ quality of life.

## CONCLUSION

The Brazilian Portuguese version of the Quality of Life Questionnaire Vascular Access Device presents adequate validity and reliability indicators, supporting its application to Brazilian patients with long-term vascular access for chemotherapy.

## SUPPLEMENTARY MATERIAL

Translation and validation of the Brazilian Portuguese version of the quality of life vascular access device questionnaire for chemotherapy patients

Bruno Jeronimo Ponte, Carolina Carvalho Jansen Sorbello, Ricardo Ferreira Mendes de Oliveira, Maria Fernanda Cassino Portugal, Andressa Cristina Sposato Louzada, Marcelo Fiorelli Alexandrino da Silva, Lucas Lembrança Pinheiro, Cynthia de Almeida Mendes, Nelson Wolosker

**DOI: 10.31744/einstein_journal/2025AO1665**

**Qualidade de vida - questionário sobre dispositivos de acesso vascular - versão em português**

Estamos tentando verificar o quanto o seu cateter tipo Hickman, Port-a-Cath ou PICC interfere em sua vida.

Por favor, tire alguns minutos para responder às questões a seguir.

Por favor, indique sua resposta circulando o número que melhor se aplica à sua rotina.

Obrigado pela sua ajuda!

Por favor indique a data de realização do questionário: _____ /_______/________

O cateter reduz sua habilidade de completar as seguintes atividades do dia-a-dia?

**Table:**

	Nem um pouco	Um pouco	Bastante	Muito
Dirigir um veículo?	1	2	3	4
Sair ou entrar em um veículo?	1	2	3	4
Usar o transporte público?	1	2	3	4
Ir às compras?	1	2	3	4

O cateter afeta sua habilidade para realizar atividades normais do dia-a-dia, como:

**Table:**

	Nem um pouco	Um pouco	Bastante	Muito
Comer	1	2	3	4
Higiene - tomar banho, tomar duchas, pentear o cabelo, se secar, etc?	1	2	3	4
Dormir	1	2	3	4
Mobilidade ou deslocamento?	1	2	3	4
Atividade normal do trabalho?	1	2	3	4
Atividade física: nadar, etc.	1	2	3	4
Hobbies: cuidar das plantas, desenhar, etc	1	2	3	4
O cateter te deixa envergonhado?	1	2	3	4
O cateter te atrapalha a socializar?	1	2	3	4
Você sente medo de infectar seu cateter?	1	2	3	4
Você sente medo de estragar seu cateter?	1	2	3	4
Até que ponto você acha que o cateter tem um impacto negativo na sua qualidade de vida?	1	2	3	4

Por favor, use a caixa abaixo para escrever qualquer observação adicional que você acha relevante sobre seu cateter e o impacto na sua qualidade de vida:

## References

[1] Wolosker N, Yazbek G, Nishinari K, Malavolta LC, Munia MA, Langer M (2017). Totally implantable venous catheters for chemotherapy: experience in 500 patients. J Vasc Bras.

[2] Brasil. Ministério da Saúde (2023). Instituto Nacional de Câncer (INCA). Estimativa 2023 - Incidência de Câncer no Brasil.

[3] Zerati AE, Wolosker N, de Luccia N, Puech-Leão P (2017). Cateteres venosos totalmente implantáveis: histórico, técnica de implante e complicações. J Vasc Bras.

[4] Wolosker N, Yazbek G, Munia MA, Zerati AE, Langer M, Nishinari K (2004). Totally implantable femoral vein catheters in cancer patients. Eur J Surg Oncol.

[5] Wu O, McCartney E, Heggie R, Germeni E, Paul J, Soulis E (2021). Venous access devices for the delivery of long-term chemotherapy: the CAVA three-arm RCT. Health Technol Assess.

[6] Patel GS, Jain K, Kumar R, Strickland AH, Pellegrini L, Slavotinek J (2014). Comparison of peripherally inserted central venous catheters (PICC) versus subcutaneously implanted port-chamber catheters by complication and cost for patients receiving chemotherapy for non-haematological malignancies. Support Care Cancer.

[7] Taxbro K, Hammarskjöld F, Thelin B, Lewin F, Hagman H, Hanberger H (2019). Clinical impact of peripherally inserted central catheters vs implanted port catheters in patients with cancer: an open-label, randomised, two-centre trial. Br J Anaesth.

[8] Leiderman DB, Souza KP, Binatti CE, Mendes CA, Teivelis MP, Brito CF (2021). Arm mobilization provokes deformity of long-term indwelling ports implanted via the jugular vein. J Vasc Surg Venous Lymphat Disord.

[9] Pu Y, Lou Li ZS, Zhi XX, Shi YA, Meng AF, Cheng F (2020). Complications and Costs of Peripherally Inserted Central Venous Catheters Compared with Implantable Port Catheters for Cancer Patients: Meta-analysis A. Cancer Nurs.

[10] Moss JG, Wu O, Bodenham AR, Agarwal R, Menne TF, Jones BL, Heggie R, Hill S, Dixon-Hughes J, Soulis E, Germeni E, Dillon S, McCartney E, CAVA trial group (2021). Central venous access devices for the delivery of systemic anticancer therapy (CAVA): a randomised controlled trial. Lancet.

[11] Ryan C, Hesselgreaves H, Wu O, Moss J, Paul J, Dixon-Hughes J (2019). Patient acceptability of three different central venous access devices for the delivery of systemic anticancer therapy: a qualitative study. BMJ Open.

[12] Parás-Bravo P, Paz-Zulueta M, Santibañez M, Fernández-de-Las-Peñas C, Herrero-Montes M, Caso-Álvarez V (2018). Living with a peripherally inserted central catheter: the perspective of cancer outpatients-a qualitative study. Support Care Cancer.

[13] Kang J, Chen W, Sun W, Ge R, Li H, Ma E (2017). Health-related quality of life of cancer patients with peripherally inserted central catheter: a pilot study. J Vasc Access.

[14] Fonseca IY, Krutman M, Nishinari K, Yazbek G, Teivelis MP, Bomfim GA (2016). Brachial insertion of fully implantable venous catheters for chemotherapy: complications and quality of life assessment in 35 patients. einstein (São Paulo).

[15] Ferreira PL, Ferreira LN, Pereira LN (2013). Contributos para a Validação da Versão Portuguesa do EQ-5D. Acta Med Port.

[16] Koo TK, Li MY (2016). A Guideline of Selecting and Reporting Intraclass Correlation Coefficients for Reliability Research. J Chiropr Med.

[17] Ritti-Dias RM, Gobbo LA, Cucato GG, Wolosker N, Jacob W, Santarém JM, Carvalho CR, Forjaz CL, Marucci Mde F (2009). Translation and validation of the walking impairment questionnaire in Brazilian subjects with intermittent claudication. Arq Bras Cardiol.

[18] Varella AY, Fukuda JM, Teivelis MP, Campos JR, Kauffman P, Cucato GG (2016). Translation and validation of Hyperhidrosis Disease Severity Scale. Rev Assoc Med Bras.

[19] (2019). States Valuation of EQ-5D-5L Health States Using an International Protocol. Value Health.

